# Supplementary material for: Factors affecting the mental health of pregnant women using UK maternity services during the COVID-19 pandemic: a qualitative interview study
Source: BMC Pregnancy Childbirth. 2022 Apr 12;22:313. doi: 10.1186/s12884-022-04602-5 (PMC9005019; doi:10.1186/s12884-022-04602-5)
Supplement: Supplementary file 1 — Additional file 1. [file 12884_2022_4602_MOESM1_ESM.docx]

# Supplementary File - Adult Maternity Service User group

**Please note – Only questions that appear in BOLD below were asked during all interviews. This list contains many optional prompts that were used variably.**

**Ask to describe ‘normal life’**

- Employed? Type of job, hours etc,
- Education/study
- Full time parent or carer?
- Use of any community services?
- Who you normally live with, does this change, separated/ extended family?
- If they have a long-term condition/cancer they mentioned (what condition, when diagnosed, if on or whether they have had complete treatment)
  - What was current treatment plan? How was it being managed? What was usual routine for appointments/follow-up?
- Whether you would usually have done any type(s) of regular exercise (whatever they perceive as exercise including walking/gardening)

UNDERSTANDING AND ADHERENCE TO GUIDELINES

**At the moment, are you self-isolating (how long for, reasons for this) a key worker, working but not a key worker, social distancing/ ‘staying at home’**

- Please describe what this is for you and your family/ household?
  - i.e. are you self-isolating with neighbours helping to get groceries, or going out for these?
  - Or self-isolating with no outside exercise? Etc – if self-isolating without outside exercise how are you feeling about this? Do you have a garden or outdoor space?

**PREGNANCY AND MATERNITY CARE**

**Can you tell me more about your experience of pregnancy during the Covid-19pandemic?**

Prompts include:

- Was this your first pregnancy? If not, how does this experience compare to previous pregnancies?
- What has been the impact on any normal appointments? (cancelled, delayed, unable to speak to appropriate healthcare professional, changed to different method/location of appointment e.g. online/telephone)
- What has been the impact on any other maternity services you would have otherwise used? (cancelled, delayed, changed from usual treatment plan, unable to get medication/prescriptions)
- Changes to birth plan?
- What has been the impact on any other services or groups you would have otherwise used? (e.g. NCT or support groups for pregnant women)
- Did you have any specific worries about your pregnancy because of the pandemic?
- Have you been diagnosed with COVID-19 during your pregnancy, or suspected you might have had COVID-19 whilst pregnant? Did this effect your experience of/access to services?
- How have you felt about [any mentioned changes/impact above]?

**SOCIAL LIFE**

**How would you describe your social life now that social distancing measures have been brought in because of Covid-19? Please tell us about this**

Prompts include:

- How would you describe your social network – for example size, types of people, types of relationships, do they live with you, nearby or further away, how often do you see each other, how well do you know each other? How do you interact, face to face, online or social media?
- Social activities?
- Could you describe any community services/participation or volunteering participation?
- Could you describe the social support you have? (such as emotional support, advice and information, someone to help you with money or milk/bread/essentials, getting medication/access to healthcare, community services)
- Can you tell us about any ways your social networks/ friendship groups influence you, such as peer pressure, or encouraging you to get involved in things? Do you compare your life to theirs?
- Social engagement (social roles, bonding, attachment)
- Have your experiences of pregnancy/becoming a new mum during the pandemic had any impact on your social life?

MENTAL HEALTH

**How do you feel about the changes that have been brought about by Covid-19?**

**Have they had any impact on your mental health or wellbeing? Please tell us about these**

- What are the things most bothering you at the moment?
- Have you experienced any impact on positive emotions? (prompts: how deeply you can engage with what you are doing, sense of meaning/ purpose, relationships with others, how well you are managing and feelings of control over your situation?)
- Has there been any impact on your sense of identity?
- Have you experienced any negative psychological feelings? (prompts: such as shame, guilt, lack of pleasure, anxiety, worry)
- Have you been doing/ planning anything to help with this?
- Please tell us about any physical symptoms due to being stressed or anxious? (prompts: fatigue, sleep problems, pain, illness symptoms, palpitations)
- Have your experiences of pregnancy/becoming a new mum during the pandemic had any impact on your mental health/wellbeing?

PROSPECTION

**Has the pandemic meant that you have any worries for the future?**

**How are these different from the worries you had before?**

- Sense of control/ powerlessness
- Severity of worries / perspective

**Will this change the way you live your life in future?**

- The way you connect with others
- How you look after yourself
- How you support others
- How you work?
- How you exercise?

Has this changed any of your priorities for the future?
