# Supplementary material for: Factors affecting the mental health of pregnant women using UK maternity services during the COVID-19 pandemic: a qualitative interview study
Source: BMC Pregnancy Childbirth. 2022 Apr 12;22:313. doi: 10.1186/s12884-022-04602-5 (PMC9005019; doi:10.1186/s12884-022-04602-5)
Supplement: Supplementary file 2 — Additional file 2. [file 12884_2022_4602_MOESM2_ESM.docx]

Appendix 2 – Examples of full quotes from research participants

| Stage of Pregnancy | Theme | Subtheme | Quote Examples from Transcripts |
| --- | --- | --- | --- |
| Pregnancy | **1. Some pregnancy discomfort alleviated by social distancing measures** | 1.1 Avoidance of unwelcome attention from others  1.2 Better management of health and wellbeing through staying at home | *“I did not see anybody; nobody touched my bump. I remember being driven insane in my first pregnancy. Because my manager, who is very lovely… Used to touch my bump, every time she saw me, without asking.”*  *“I think in a way not being able to see people has helped me a little bit. I think there have been times when I’ve been really, really low in the last year and a half and it’s been nice not to have to go and pretend that I’m all right… it’s been really nice not seeing anyone and just keeping it low key.”*  *“I found it useful because I was very, very tired all the time, and so being able to work from home and sleep in between getting work done was really helpful.”*  *“I would throw up and then I would just carry on, or I would be very tired, and I would lie down and then I would go back to work… And I was thinking, if it had been normal times, I would have had to take loads of time sick, off work, because I wouldn’t have been able to face getting on a train. The time I would have been on a train, is the time I threw up every day…”*  *“There was some benefits to social distancing, and like I said, we all benefited in terms of not getting sick.”*  *“I would say on the pregnancy, I think it had a positive impact on my mental health. I think that I always would have been anxious, I always would have been anxious about the health of the baby, but the fact that I could work from home and that I could exercise every day, and I could eat my own food, in my own house, and that I didn’t have to go anywhere.”* |
| Pregnancy and Parenthood | **2. Importance of relationships that support coping and adjustment** | 2.1 More time to build a connection as a family  2.2 Benefits of the support bubble system  2.3 Importance of parent groups for support | *“I mean the only one positive thing for me out of this whole COVID thing… Was that I could spend time, I got to spend a lot of time with my children at this age where they're talking and really engaging. Because maternity leave you have the baby and then you go back to work when all the exciting stuff starts to happen. So, it was really nice to be at home and spend that time with the children. Because you would never get that opportunity again. But that is the only thing for me that was positive. There's nothing else at all.”*  *“It’s certainly changed who I am in some ways. And it’s made me much more comfortable to be at home, it’s slowed down my pace of life in a way I would never have chosen to do, but actually has been really beneficial. We’ve spent much more time together just at home as a family, enjoying ourselves.”*  *“It has made us reassess what's important and neither me or my husband think that we're ever going to go back to work in an office full time. He might go maybe three days a week, I might go two. It makes it cheaper for nursery.”*  *“The lockdown and the pandemic has meant that my partner’s working from home, and the support that he provides me, I wouldn’t have had if he was at work. So, swings and roundabouts, I guess, a little bit, in terms of not getting support, maybe, from my wider social network, but having the support of my partner has been invaluable.”*  *“It just seemed incredibly unfair that my husband was allowed to go to work, we had a new baby, and go and do all of these dangerous thing. But I couldn’t have my mum round for a coffee to help me out.”*  *“One of my friends who had actually had a miscarriage herself a few weeks earlier did come over and dropped off some medication and sat with me in the garden and had a chat. Which I don’t know if that was technically allowed then but I wasn’t really thinking about that.”*  *“From December, the rules were that you could form a support bubble if you had a child under 1. And I just think that should have come in so much sooner ‘cause you just need that support – you know, me and my mum have never been really close, but when I became pregnant I suddenly really needed her.”*  *“I was really lucky to attend a lockdown baby group at our children’s centre, so even, obviously, when we had the restrictions, at least that didn’t stop. So, that was good, being able to at least, to me, to just have that one-hour space to bounce off one another, which we did.”*  *“I did some of the things online, but most of it, I just thought, you know what, I’d rather be there in person. Socially, not having those groups meant that my mental health was worse than it would have been, I think, otherwise.”*  *“We did NCT over Zoom. I can’t say I found it that useful, I found it quite difficult to form the relationships with the other people over Zoom.”*  *“And you also felt like, even if you went out to the park or to a walk, you’re distant and wary of everybody else around you, so you don’t really see or talk to anybody new. So, yes, the impact of just not forming new friendships with other new parents, or as a first-time mum, just missing that opportunity with would have happened from the start of having your child, it’s just missed.”* |
|  | **3. Missed pregnancy and parenthood experiences** | 3.1 Grief for a missed pregnancy experience  3.2 Sadness that pregnancy and parenthood could not be shared with others | *“And I felt like, I still feel a bit like, I had to grieve what I lost, like I always imagined what it’d be like to be pregnant.”*  *“Weird little things that I was really quite looking forward to, like a baby shower.…pram shopping, it feels so superficial, but it's actually quite a nice ritual to go through. It's like going to pick out baby clothes and find the pram and look at cribs, we never really got to do that.”*  *“I’ve waited 37 years to have a baby. I had one in the middle of a pandemic and all the normal things were taken away from me. I felt really sad about it, and scared.”*  *“I think it was quite hard, actually, being pregnant for the first time and then not seeing people, and people not seeing my bump growing and things like that. I think that was quite a big mental impact on me. And I think it felt like, I don’t know, it wasn’t happening, in a way. It was a bit strange. There were obviously pros to being at home, but I think not seeing people and not having that normal journey through your pregnancy, socially.”*  *“It kind of felt like a secret, being pregnant, ‘cause I didn’t see anyone, and no one apart from the medical staff and my parents saw me, and it’s kind of like everyone was so caught up in having to adjust with what’s around them and the evolving state of the world, that I kind of felt forgotten about.”*  *“I haven’t been able to meet up with my friends and family and share early motherhood with them. Our babies changed so quickly, don’t they, that I feel I’ve had to grieve on what I’ve missed out on and what he’s missed out on. “*  *“It’s heart-breaking, ‘cause I’ve not been able to have people around and he’s not been able to have people around for that support. Especially with [husband’s name] working, you know… the days are long and the days are lonely.”* |
| Birth | **4. Mental health consequences of birth partner and visitor restrictions** | 4.1 Upset about partners being excluded from the healthcare experience  4.2 Stress of decision making and help seeking without partners present | *“I think that was really difficult, especially after having a miscarriage and going in and hearing the news about how your pregnancy is going all on your own, I did, on multiple occasions, ask if I could have him on video, and the first time they said no, the second time, I think at the 24-week scan, they said yes, and I called him towards the end of the scan, when they were telling us the sex of the baby. So, that was really weird.”*  *“I still had to go into the hospital [after miscarrying] but I would have to go in and go through that process alone and my husband wasn’t allowed into the hospital or into the waiting room even. So, that was really awful because I was not in a great state… The whole thing you’re going through when your body is losing something as well, it’s so traumatic on your body... Going to the hospital then alone and being alone through that process and coming out of there alone… It just isolates the two of you even more which is the last thing you need really.”*  *“From my perspective actually, it was just a bit limited in terms of face-to-face time, whereas for him there was nothing, he wasn’t involved essentially, that was probably the worst thing about it. It’s our first baby…”*  *“I said, ‘can I ring my husband, whilst we’re in the appointment?’ And they were like, ‘you can't video call’… Okay, that's weird, but if that’s what you want. They said, ‘can you confirm you're not going to record us?’… I remember being so insulted. Why would I record you, and even if I did, what skin is it off your nose?”*  *“That was really upsetting, being on the maternity ward myself, my waters breaking, getting really scared because they were doing a staff turnover when it all happened and I couldn’t get anyone’s attention… if he’d been there he could have gone off and got someone for me, and it was all just a blur really… And it’s such a traumatic thing for your body to go through isn’t it, giving birth, but, you just need your birth partner there. Whether that’s your partner, or your mum, or your friend, whatever, just someone to bring you down to that level of coping – it’s really important, and that’s what so many women have missed out on.”*  *“I'd been admitted to this observation ward. And then, because they didn’t have a midwife available, whether that is because of COVID or what, I couldn’t be put on the labour ward initially. I did a lot of it on my own, because he couldn’t come up.”*  *“So, from the Monday onwards, I couldn’t have any visitors, so obviously, that was then challenging … my partner ended up outside the hospital, being told he couldn’t come in, which was very traumatising on both parts.”*  *“[The baby] had reduced movements once and I had to go into triage by myself. And then, when I was 39 weeks and she wasn't moving again, I had to go in and they were like, well, we would recommend induction. I had to go and find [my birth partner] outside in the hallway and have a conversation. He couldn't be in there to talk to the doctor about a really pretty important decision that we had to take. I think he felt a little bit bulldozed by it. Because I'd heard all the information, but then, of course, I couldn't really relay it.”*  *“And the other thing was not having your partner there for any of the antenatal clinics or any of the times I had to go into hospital for check-ups... And I think that it was quite hard to make choices on things by myself. And quite hard to have a voice. And I think that your birth partner or your partner throughout the whole thing, it’s quite important to have them there for you to have that voice, because sometimes, when you’re in pain or you’re upset or you’re feeling a bit vulnerable, you can’t actually articulate what you really need or what you want.”*  *“I was really scared I’d be there for days on my own in pain without my husband, or that it would suddenly happen really quickly and he wouldn’t be there, and he’d miss the birth of the baby. So it was quite a major factor in me deciding to ask for a C-section.”*  *“So, the whole, actual giving birth experience, the medical bit, was great, and then the post-natal bit was just awful, it was so horrible and frightening…I realised that I was hallucinating… I couldn’t sleep, because, not only was my baby awake, but all the others were, at various points, as well… on the second day my husband came to visit, I got him to go and say, look, my wife has mental health issues, please can you give her more support.”* |
| Across the care pathway (pregnancy, birth and postnatal) | **5. Maternity services under pressure** | 5.1 Emotional impact of staff shortages  5.2 Staff misinformation about social distancing rules | *“… You can’t even sit in a waiting room with other people. You’re waiting for a slot to see somebody and they were all so busy in the hospital that you could be in a waiting room for such a long time on your own just waiting to be seen by someone.”*  *“Lots of staff were off sick, and I think the hospital was in a state of chaos… the hospital were under a lot of stress, but it was just the post-natal stuff… the bit about caring for the baby, there was just no help at all, really.”*  *“I was very disappointed by the level of care that was unfortunately provided to me, because it didn’t help that I couldn’t have anybody there, so there was just, obviously, me and baby, and I felt that the basic needs, like making sure that they’ve given me a bed bath, or support me to go and use the shower or supporting me to get changed, or any of that, just didn’t happen, whatsoever.”*  *“I felt like, to some extent, they might not be following up things they would normally follow up or perhaps dealing with things with the same urgency that they normally would, because of COVID.”*  “*So, the first appointment was lovely, and really reassuring and felt really positive, and then after that it kind of went downhill quite quickly, and I think that was because it was a bit haphazard. But I think it was probably also haphazard for the people trying to deliver those services, they didn’t sound like they were having an easy ride at all. So yes, it was just difficult I suppose not to get frustrated, and just try being mindful of the fact that they don’t know what’s going on as much as you, probably.”*  *“I remember contacting the assisted conception unit in the hospital and them saying, ‘it’s really difficult here, we’re short-staffed and we’ve got COVID happening, this is the emergency.’ So, you feel really bad about asking for support. You realise that no one’s dying by at the same time, oh my God, emotionally you feel like you’re dying. I know that’s quite dramatic to say but you feel so low and then you feel like you can’t contact anybody. So, it compounds that feeling.”*  *“I went by myself, and the entrance, it said, if this is your first appointment at the foetal medicine unit, your husband can come, your partner can come. I asked them, can my partner come? They were, ‘we don’t know, they keep changing the rules, none of us has got any idea.’ So, they went and asked, and in the end they were like, just send him up.”*  *“I think there was some confusion with the doctors and the nurses around what the policies were, not that they told me that, but that’s what I felt. And then, when I had more of an understanding of what the policies were, when I was a bit more, like, this is what I’m allowed to do, then I think they gave in a bit more.”* |
|  | **6. Lack of connection with staff** | 6.1 Communication difficulties  6.2 Prevention of touch due to COVID-related restrictions  6.3 Disruptions to continuity of care | *“That was really weird, and just going in with masks and seeing the doctors and the nurses through masks, that was all really weird. I’m quite a social person and I chat, it just made it all a lot more difficult.”*  *“And I know that, normally, the health visitors would see you once a month… that is something that I really felt was quite a worry for me, especially in the beginning, because my son was premature and I was concerned about his weight and concerned about just lots of things developmentally. And I think just having a phone call about that was quite concerning.”*  *“The pregnancy didn’t show very visibly on me… I had no bump at all, really, for about five and a half months. So, I was always quite anxious, ‘is the baby developing properly?’… because all the midwife appointments were on the phone, it was probably only about five months in, where I actually got measured, and they were like, oh, yes, that’s fine.”*  *“Post-natal, I think it’s a six-week check for mum. That didn’t happen in person, and that was, for me, a really big issue, because my C-section scar was infected… nobody was able to check it after, to make sure that it was okay, and it would have normally happened at the six-week appointment. But because that happened over the phone, they weren’t able to have a look at it. So, I think that was, personally, that should have been an appointment that happened face-to-face.”*  *“I think it was just difficult in terms of, some of the midwives in the hospital, the advice is not to touch the babies so much. And I think when we were at home, you’ve still got someone coming in, and wearing a mask. It couldn’t be as personal, maybe, or interactive as what it might have been.”*  *“[My baby] was crying, and none of the nurses were able to pick him up. I was pulling on my trousers, and I had him in my hand, and I couldn’t even pass him over to a doctor, to anyone, so I had to put him back in the buggy, and he was crying, I was trying to change. It was just complete madness, and you could see that the nurses were looking at me, quite sympathetic, but they couldn’t do anything.”*  *“I had to tell my story every time, that was just really distressing, and none of them read the notes in advance…I cannot describe how stressful my pregnancy was, and it was definitely compounded by having no-one hold my hand through it. And of course, now, they’ve got a policy of the same midwife for the whole pregnancy. And I’ve seen that come a bit out of the pandemic, and a bit out of people’s feedback in general.”*  *“I rarely saw my actual, my allocated midwife. Each time I went, it was someone different… I couldn’t build that rapport when it’s not the same person every time. So, yeah that was tough.”*  *“Postnatally… because it was different people coming after the birth, there wasn’t any, I don’t know, I guess it didn’t feel, It felt a bit distant. It didn’t feel very supportive as I guess it could’ve been.”*  *“I would say from 36 weeks onwards, I was seeing a midwife nearly every week, and I was seeing the same midwife, and that really made a difference, with a student, who was really good as well. I felt far more supported, because I was like, I’m seeing, I know the midwife’s name, I’m going to her next week, I’ll save up this question. She’s making sure that everything’s okay, she’s feeling the baby move, listening to his heartbeat, all that kind of stuff, and I felt far more assured.”* |
